# Integrated radiomics and deep learning model for identifying medullary sponge kidney stones

**DOI:** 10.3389/fmed.2025.1623850

**Published:** 2025-07-25

**Authors:** Yubao Liu, Haifeng Song, Daxun Luo, Rui Xu, Zheng Xu, Bixiao Wang, Weiguo Hu, Bo Xiao, Gang Zhang, Jianxing Li

**Affiliations:** Department of Urology, Beijing Tsinghua Changgung Hospital, School of Clinical Medicine, Tsinghua University, Beijing, China

**Keywords:** deep learning, radiomics, medullary sponge kidney, kidney stone, artificial intelligence

## Abstract

**Background:**

Medullary sponge kidney (MSK) is a rare congenital anomaly frequently associated with nephrolithiasis. Accurate preoperative differentiation between MSK stones and non-MSK multiple kidney stones remains challenging, yet it is essential for effective clinical decision-making. This study aims to develop a novel diagnostic model that integrates radiomics and deep learning features to improve the differentiation of MSK stones using CT imaging.

**Methods:**

This single-center, retrospective study included patients who underwent surgical treatment for multiple kidney stones at Beijing Tsinghua Changgung Hospital between 2021 and 2023. All MSK and non-MSK cases were confirmed via endoscopic surgery. Radiomics features were extracted from manually delineated regions of interest (ROI) on nephrographic-phase CT images, while deep learning features were derived from a ResNet101-based model. Three diagnostic signatures—Radiomics (Rad), Deep Transfer Learning (DTL), and Deep Learning Radiomics (DLR)—were developed. A Combined model was constructed by integrating clinical variables with DLR features to further enhance diagnostic accuracy. Model performance was evaluated using AUC, calibration curves, Net Reclassification Index (NRI), and Integrated Discrimination Improvement (IDI) analyses. Additionally, Gradient-weighted Class Activation Mapping (Grad-CAM) visualization was employed to identify imaging regions critical to classification, improving interpretability.

**Results:**

A total of 73 patients with multiple kidney stones were analyzed, comprising 34 MSK cases and 39 non-MSK cases, encompassing 110 kidneys in total. The DLR signature demonstrated high diagnostic accuracy, with AUCs of 0.96 in both the training and test cohorts. The Combined model further enhanced diagnostic performance, achieving AUCs of 0.98 in the training cohort and 0.95 in the test cohort. Calibration curves indicated strong agreement between predicted probabilities and observed outcomes. Furthermore, NRI and IDI analyses highlighted the superior predictive power of both the DLR and Combined models compared to other approaches.

**Conclusion:**

This study introduces an innovative approach for MSK stone diagnosis by integrating radiomics and deep learning features. The proposed model offers high diagnostic accuracy and promising clinical utility.

## Introduction

Medullary sponge kidney (MSK) is a rare congenital renal anomaly characterized by cystic dilations of the collecting ducts in the renal papilla, resulting in its distinctive “sponge-like” appearance on imaging (1). These cystic dilations predispose patients to urinary stasis and alterations in the local metabolic microenvironment, often leading to clinical associations with nephrolithiasis, nephrocalcinosis, and metabolic abnormalities such as hypocitraturia and renal tubular acidosis (2, 3). These metabolic disturbances significantly increase the risk of recurrent stone formation and chronic kidney disease, with the prevalence of nephrolithiasis in MSK patients reported up to 69.6% (4).

Preoperative differentiation between MSK stones and non-MSK multiple kidney stones is critical for optimizing patient management. Unlike ordinary stone patients, MSK cases often require tailored therapeutic strategies, such as metabolic evaluation and potassium citrate treatment, to prevent recurrence and address underlying metabolic abnormalities (1). Additionally, the identification of MSK stones significantly impacts surgical planning, as the associated anatomical and functional anomalies may necessitate modifications to surgical techniques (5). Accurate and early diagnosis also helps to avoid unnecessary interventions, reducing the risk of kidney damage and chronic pain complications (6).

Diagnosing MSK stones presents long-standing challenges due to their imaging similar to other kidney stones, particularly in early-stage cases graded as Forster 1–2, where differentiation is more difficult (7). Traditionally, intravenous urography (IVU) has been considered the gold standard for MSK diagnosis, revealing the characteristic “bouquet-like” appearance caused by the dilation of the collecting ducts (2). However, high radiation exposure and low sensitivity IVU for small stones have led to its replacement by non-contrast CT in the diagnosis of urolithiasis, which has led to fewer cases of MSK being diagnosed. While CT urography (CTU) offers a modern alternative by combining anatomical and functional evaluations, its sensitivity and specificity for MSK diagnosis remain limited, especially in distinguishing MSK stones from non-MSK multiple kidney stones.

Recent advancements in radiomics and deep learning have provided new opportunities for imaging diagnostics. These techniques extract high-dimensional quantitative features (e.g., texture, shape, and intensity) from CT images, enabling the detection of subtle differences imperceptible to the human eye (8). Integrating radiomics features with deep learning algorithms has significantly extended the application values of traditional imaging approaches in various diseases, enabling the development of predictive models with improved accuracy and robustness (9–11).

This study aims to develop a novel classification model based on CTU data by integrating deep learning features with traditional radiomics features to improve the preoperative differentiation of MSK stones from non-MSK multiple kidney stones. This approach leverages the imaging strengths of CTU and optimizes diagnostic performance through artificial intelligence, contributing to personalized clinical management and the advancement of precision medicine.

## Methods

### Study design and patient selection

This single-center, retrospective study aimed to develop a predictive model based on CTU images utilizing deep learning and radiomics features to distinguish MSK stones from non-MSK multiple stones. Patients included in the study underwent surgical treatment for multiple renal stones at Beijing Tsinghua Changgung Hospital between 2021 and 2023, with all MSK cases confirmed during endoscopic surgery.

**Figure 1:**
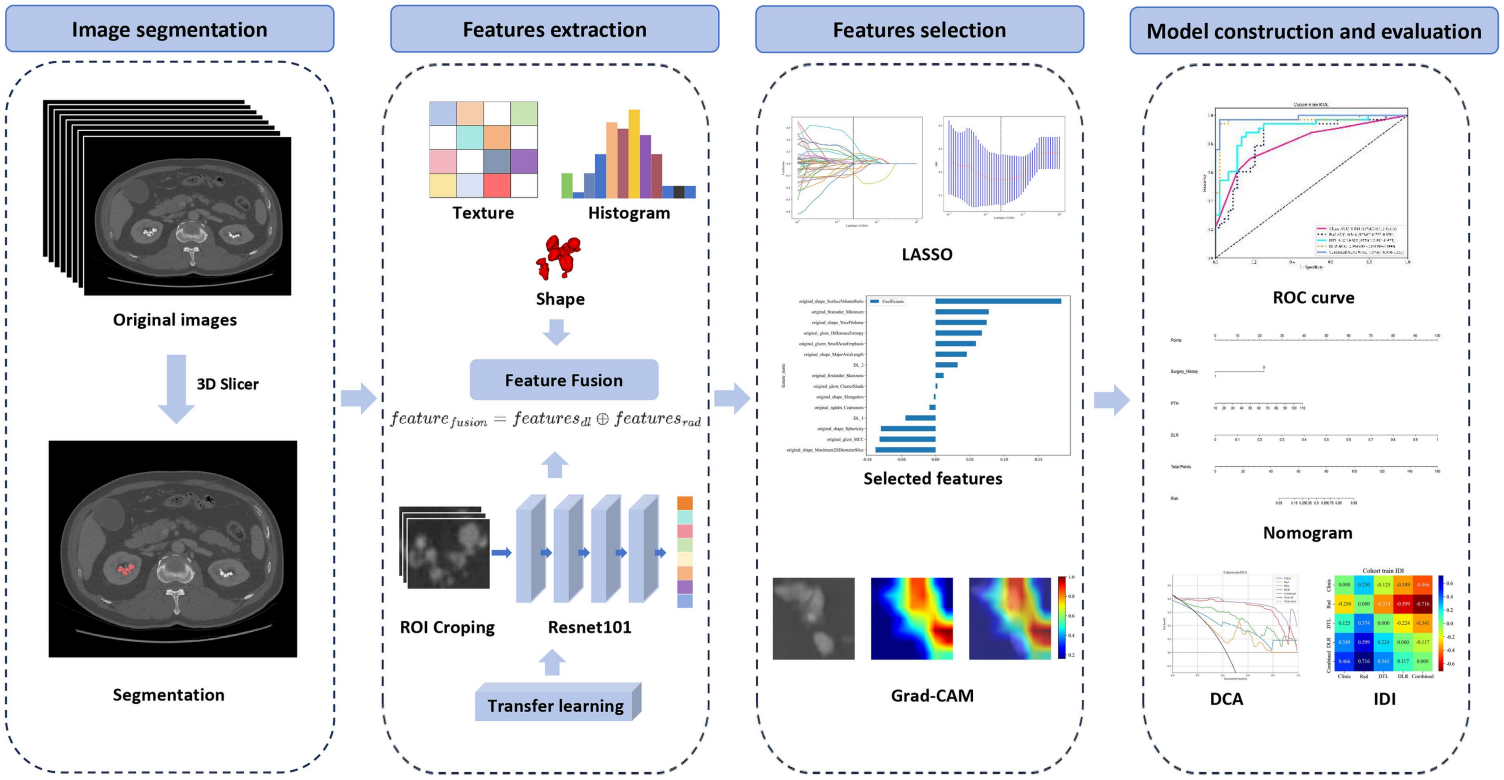
Workflow for developing a model integrating radiomics and deep learning to differentiate MSK stones from non-MSK stones. MSK medullary sponge kidney.

The inclusion criteria were as follows: patients with multiple renal stones (≥3 stones) confirmed by CT imaging and treated with endoscopic procedures, such as flexible ureteroscopy or percutaneous nephrolithotomy; availability of high-quality CTU images; and complete clinical data records. Patients were excluded if they had other renal anomalies (e.g., polycystic kidney, horseshoe kidney, and ectopic kidney) or if their images were of poor quality or data were incomplete. Patients were randomly assigned to training and validation cohorts in a 7:3 ratio for model development and performance evaluation. The flowchart of participant selection and the workflow of model development are presented in Supplementary Figure S1 and Figure 1, respectively.

### Image acquisition and preprocessing

All patients underwent three-phase contrast-enhanced CT scans (arterial, nephrographic, and excretory phases) using a Discovery CT 750 HD scanner (GE, USA). Images were acquired with a tube voltage of 100–120 kV, an automatic tube current of 200–350 mA, and a rotation time of 0.5 s. The original 5-mm slice thickness was reduced to 1.25 mm for further analysis.

Nephrographic phase images were selected for further processing. Using the 3D Slicer software (version5.6.2, https://www.slicer.org/) (12), the region of interest (ROI), specifically the stone area, was manually delineated by two experienced urologists. In cases of disagreement, a senior expert acted as the final arbitrator to ensure consistency. To evaluate the inter-observer spatial agreement of these segmentations, the Dice similarity coefficient was calculated for each case. Subsequently, a comprehensive set of radiomics features was extracted from each urologist’s segmentations. The consistency of these feature values between the two observers was then quantified using the Intraclass Correlation Coefficient (ICC, type 2,1).

### Radiomics procedure

Radiomics features were extracted from each ROI using the PyRadiomics tool (13), encompassing shape-based (2D and 3D) features (e.g., volume, surface area, and aspect ratio), first-order statistics (e.g., mean gray-level intensity and standard deviation), and texture features derived from the Gray-Level Co-occurrence Matrix (GLCM), Gray-Level Run Length Matrix (GLRLM), Gray-Level Size Zone Matrix (GLSZM), Gray-Level Dependence Matrix (GLDM), and Neighboring Gray Tone Difference Matrix (NGTDM). Feature selection was performed through a multi-step process: Z-score normalization to standardize feature values, a t-test to retain features with *p*-values < 0.05, removal of highly collinear features using Pearson correlation coefficients (correlation > 0.9), and Lasso regression to identify the most predictive features for the model.

### Deep learning procedure

#### Data preparation and model training

For each patient, the slice containing the largest ROI was selected as the representative image. To reduce computational complexity and minimize background noise, only the minimum bounding rectangle enclosing the ROI was retained for further analysis. To standardize the intensity distribution across the RGB channels, Z-score normalization was applied to the images, which were then used as inputs for the model. During the training phase, real-time data augmentation strategies, such as random cropping, horizontal flipping, and vertical flipping, were employed to improve the robustness of the model. For testing images, only normalization was applied to maintain consistency in evaluation.

#### Model training

Transfer learning was implemented to improve the adaptability of the model to diverse patient populations. The model was initialized with pretrained weights from the ImageNet database, enhancing its ability to generalize across different datasets. Learning rate adjustment was a critical component of the training process. A cosine decay learning rate strategy was adopted to optimize convergence:


$$\eta_{t}=\eta_{\min}^{i}+\frac{1}{2}(\eta_{\max}^{i}-\eta_{\min}^{i})(1+\cos(\frac{T_{\mathrm{cur}}}{T_{i}}\pi ))$$

Additionally, stochastic gradient descent was used as the optimizer, and softmax cross-entropy was selected as the loss function to facilitate accurate classification.

#### Feature extraction

The pretrained ResNet101 model was employed to extract 2048-dimensional feature vectors from the penultimate layer. To further reduce computational demands and preserve essential information, principal component analysis (PCA) was applied to the extracted features, resulting in a compact and informative feature set for downstream analysis.

### Signature construction

Radiomics (Rad) signature: Through rigorous feature selection using LASSO, radiomics features were utilized to construct risk models based on machine learning algorithms, such as Logistic Regression (LR), Extreme Gradient Boosting (XGBoost), and Support Vector Machines (SVM). The performance of each model was comparatively analyzed to identify the optimal radiomics model.

Deep transfer learning (DTL) signature: In our deep learning approach, the output probabilities computed by convolutional neural networks (CNNs) were defined as deep learning features. Feature extraction was performed from the penultimate layer of the CNN to capture high-level abstract features relevant to stone classification.

Deep learning radiomics (DLR) signature: To develop the DLR features, we employed a pre-fusion strategy by integrating radiomics and deep learning features. Using a feature concatenation method, these features were combined into a comprehensive feature set: $\text{featur}e_{\text{fusion}}=\text{feature}s_{\mathrm{dl}}⊕\text{feature}s_{\mathrm{rad}}$ .

The integrated feature set was subjected to the same feature selection and model-building processes as those applied to radiomics features.

To construct the Combined model, we performed univariable and stepwise multivariable analyses on all clinical features to identify significant predictors. These selected clinical features were integrated with predictions from the DLR model to create a linear Logistic Regression (LR) model. This model was effectively visualized using a nomogram.

### Performance evaluation

The evaluation of the performance for all constructed models was conducted through receiver operating characteristic (ROC) analysis. Specifically, the area under the ROC curve (AUC) was calculated and subsequently compared among various cohorts using the DeLong test. Net reclassification index (NRI) and integrated discrimination improvement (IDI) were calculated to compare the performance between different models. The calibration of the models was assessed using calibration curves, with the Hosmer–Lemeshow goodness-of-fit test employed to verify their reliability. Furthermore, decision curve analysis (DCA) was utilized to evaluate the clinical utility of our predictive models, facilitating an understanding of potential benefits in clinical settings.

### Statistical analysis

The normality of continuous variables distribution was assessed by the Shapiro–Wilk test. Depending on their distribution, continuous variables were compared using either t-tests or Mann–Whitney U tests, while categorical variables were analyzed using chi-square tests. Radiomics feature extraction was carried out using PyRadiomics (version 3.0.1, https://github.com/AIM-Harvard/pyradiomics). Machine learning workflows utilized Scikit-Learn (version 1.0.2, https://scikit-learn.org/). The deep learning framework was developed with PyTorch (version 1.11.0). All statistical analyses were conducted using the OnekeyAI platform (version 4.9.1) with Python (version 3.7.12, https://www.python.org/).

## Results

### Patient clinical characteristics

A total of 73 patients were included in this study, consisting of 34 patients with MSK stones and 39 patients with non-MSK multiple renal stones. Due to the rarity of MSK stones, each kidney from patients with bilateral kidney stones was treated as an independent case. As a result, a total of 110 kidneys were analyzed, comprising 52 MSK kidneys and 58 non-MSK kidneys.

The mean age of all participants was 48.66 ± 14.43 years, with 36 women and 37 men. The clinical baseline characteristics of all patients are summarized in Table 1. No significant statistical differences were observed in the clinical characteristics between the MSK and non-MSK stone groups in either the training or test cohorts.

**Table 1:** Clinical characteristics of patients in the training and testing cohorts.

Variables	Training cohort (*n* = 50)			Testing cohort (*n* = 23)		
	Non-MSK patients (*n* = 28)	MSK patients (*n* = 22)	*p*	Non-MSK patients (*n* = 11)	MSK patients (*n* = 12)	*p*
Age, Mean ± SD	46.54 ± 16.22	46.00 ± 12.92	0.900	56.91 ± 12.59	50.92 ± 12.43	0.264
BMI, Mean ± SD	24.17 ± 4.43	26.64 ± 9.55	0.230	24.39 ± 2.44	26.15 ± 3.16	0.152
Sex, *n* (%)			0.802			1.000
Female	15 (53.57)	11 (50.00)		5 (45.45)	5 (41.67)	
Male	13 (46.43)	11 (50.00)		6 (54.55)	7 (58.33)	
Spontaneous Passage History, *n* (%)			0.253			1.000
No	25 (89.29)	16 (72.73)		7 (63.64)	8 (66.67)	
Yes	3 (10.71)	6 (27.27)		4 (36.36)	4 (33.33)	
Surgery History, *n* (%)			0.278			0.680
None	13 (48.15)	14 (63.64)		7 (63.64)	6 (50.00)	
Yes	14 (51.85)	8 (36.36)		4 (36.36)	6 (50.00)	
Urine Culture, *n* (%)			0.449			1.000
Negative	11 (39.29)	11 (50.00)		4 (36.36)	4 (33.33)	
Positive	17 (60.71)	11 (50.00)		7 (63.64)	8 (66.67)	
K, Mean ± SD	8.63 ± 24.96	3.89 ± 0.44	0.379	4.01 ± 0.37	3.99 ± 0.21	0.895
Cl, Mean ± SD	106.16 ± 2.93	106.49 ± 2.29	0.669	107.26 ± 1.98	106.47 ± 2.56	0.416
CO2, Mean ± SD	24.77 ± 3.23	24.99 ± 3.38	0.823	25.48 ± 2.80	24.93 ± 2.75	0.636
Scr, Mean ± SD	75.63 ± 23.07	84.42 ± 26.65	0.218	83.02 ± 20.85	75.83 ± 19.17	0.399
Ca, Mean ± SD	2.27 ± 0.11	2.29 ± 0.10	0.489	2.28 ± 0.10	2.23 ± 0.07	0.231
Uric acid, Mean ± SD	333.82 ± 93.03	319.18 ± 84.65	0.568	402.64 ± 112.97	355.11 ± 95.06	0.286
Hb, Mean ± SD	130.79 ± 19.66	138.27 ± 20.03	0.191	129.27 ± 18.43	136.92 ± 17.36	0.317
PTH, M (Q₁, Q₃)	41.49 (29.98, 49.13)	47.09 (33.62, 48.54)	0.475	43.15 (32.75, 48.00)	47.09 (44.87, 48.95)	0.281
24 h Ca, M (Q₁, Q₃)	5.53 (3.66, 5.53)	4.05 (3.59, 6.22)	0.670	3.25 (1.94, 5.53)	4.25 (3.87, 7.47)	0.206
24 h K, M (Q₁, Q₃)	39.05 (36.08, 39.06)	32.37 (24.27, 41.59)	0.090	39.05 (29.23, 39.05)	34.04 (30.21, 37.08)	0.558
24 h Na, M (Q₁, Q₃)	149.18 (128.05, 149.18)	147.81 (127.88, 183.02)	0.627	143.40 (89.56, 149.18)	155.99 (137.27, 226.31)	0.052
24 h UA, M (Q₁, Q₃)	3314.48 (2672.50, 3314.48)	2953.32 (2255.20, 3261.10)	0.181	3314.48 (2530.75, 3314.48)	2988.50 (2650.35, 3585.27)	0.734
24 h P, M (Q₁, Q₃)	15.54 (14.87, 15.54)	16.18 (13.54, 21.69)	0.482	15.54 (11.05, 15.91)	16.60 (14.33, 19.80)	0.229
24 h Cl, M (Q₁, Q₃)	123.46 (113.97, 123.46)	113.37 (99.65, 149.46)	0.937	123.46 (97.44, 126.43)	128.75 (117.75, 178.73)	0.116
Urine PH, M (Q₁, Q₃)	6.50 (6.00, 6.50)	6.50 (6.00, 6.50)	0.976	6.50 (6.25, 7.00)	6.25 (6.00, 6.62)	0.340

MSK, medullary sponge kidney; BMI, body mass index; Scr, serum creatin; Hb, hemoglobin.

### Radiomics and deep learning signature

To evaluate inter-observer consistency in manual ROI segmentation, we calculated the Dice similarity coefficient across 20 randomly selected kidneys. The mean Dice coefficient was 0.91, indicating excellent spatial agreement. Furthermore, ICC analysis was conducted on radiomics features extracted from the independently drawn ROIs. A total of 107 radiomics features were extracted from both sets of ROIs. Among them, 82 features (76.6%) had an intraclass correlation coefficient (ICC) > 0.75, and 71 features (66.4%) had ICC > 0.90, indicating good to excellent reproducibility.

After feature selection, a total of 15 radiomics features were included in the construction of the signature (Supplementary Figure S2A). Among various machine learning algorithms evaluated, SVM was ultimately chosen for signature construction and subsequent comparisons. The Rad signature achieved an AUC of 0.85 (95% CI: 0.76–0.94) in the training cohort and 0.79 (95% CI: 0.63–0.95) in the test cohort (Supplementary Figure S2B).

For the deep learning-based signature, 8 deep learning features were included (Supplementary Figure S2C). This DLT signature outperformed the radiomics signature, achieving an AUC of 0.90 (95% CI: 0.83–0.97) in the training cohort and 0.82 (95% CI: 0.67–0.97) in the test cohort (Supplementary Figure S2D).

To explore the recognition capabilities of deep learning models across different samples, we employed the Grad-CAM technique for visualization. Supplementary Figure S2E illustrates the use of Grad-CAM to highlight activations in the final convolutional layer relevant to classification. This approach helps identify the specific image regions that significantly influence the decision-making process of the model, thereby enhancing our understanding of its interpretability.

### DLR

**Figure 2:**
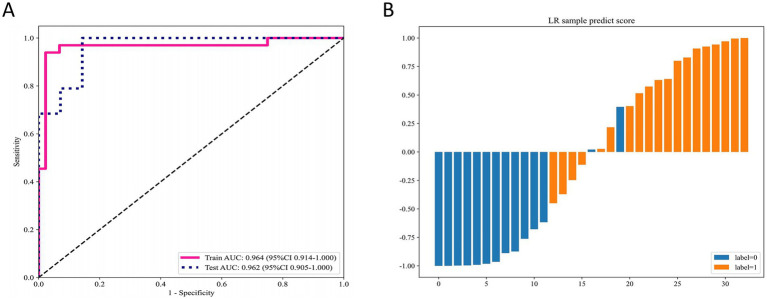
Performance of the DLR signature for differentiating MSK stones from non-MSK stones. **(A)** The ROC curves of the DLR signature in the training and testing cohorts. **(B)** Predicted scores for individual samples generated by the DLR signature. DLR deep learning radiomics, MSK medullary sponge kidney, ROC receiver operating characteristic.

After integrating and selecting radiomics features and deep learning features, a total of 13 radiomics features and 2 deep learning features were included in the construction of the DLR signature. The DLR signature was developed using a logistic regression (LR) approach. In the training cohort, the DLR signature achieved an AUC of 0.96 (95% CI: 0.91–1.00), and in the test cohort, an AUC of 0.96 (95% CI: 0.91–1.00) (Figure 2A). Figure 2B displays the predicted scores for individual samples. This separation reflects the high predictive accuracy of the DLR signature and its capability to distinguish between MSK stone and non-MSK stone effectively.

### Model comparison

**Figure 3:**
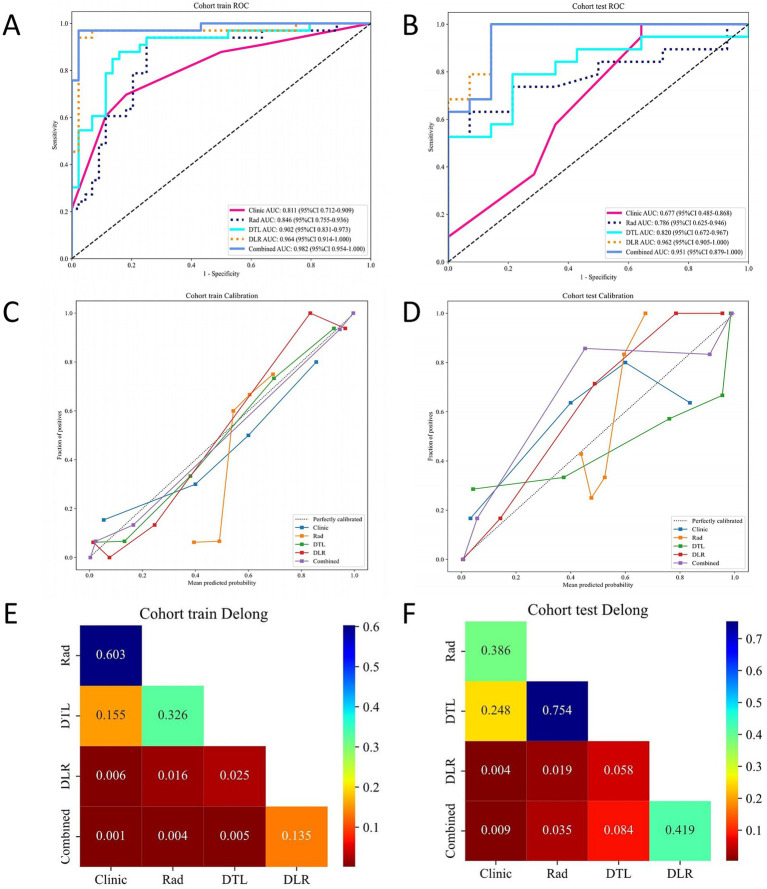
Performance comparison of different models (clinical, Rad, DTL, DLR, and Combined) in the training and testing cohorts. **(A)** ROC curves in the training cohort. **(B)** ROC curves in the testing cohort. **(C)** Calibration curves in the training cohort. **(D)** Calibration curves in the testing cohort. **(E)** DeLong test results for the training cohort. **(F)** DeLong test results for the testing cohort. Rad radiomics, DTL deep transfer learning, DLR deep learning radiomics, ROC receiver operating characteristic.

Univariate analysis did not identify any clinical features with statistically significant differences. However, we selected the two clinical variables that were closest to achieving statistical significance and combined them with the DLR model. The resulting model achieved an AUC of 0.98 (95% CI: 0.95–1.000) in the training cohort (Figure 3A) and 0.95 (95% CI: 0.88–1.00) in the test cohort (Figure 3B). The metrics of all signatures are summarized in Table 2.

**Table 2:** Metrics of performance of different signatures.

Metric parameter	Clinical		Rad		DTL		DLR		Combined	
	Train	Test	Train	Test	Train	Test	Train	Test	Train	Test
Accuracy	0.77	0.70	0.82	0.73	0.84	0.76	0.95	0.91	0.96	0.91
AUC	0.81	0.68	0.85	0.79	0.90	0.82	0.96	0.96	0.98	0.95
	(0.71–0.91)	(0.49–0.87)	(0.76–0.94)	(0.63–0.95)	(0.83–0.97)	(0.67–0.97)	(0.91–1.00)	(0.90–1.00)	(0.95–1.00)	(0.88–1.00)
Sensitivity	0.61	0.95	0.91	0.58	0.85	0.74	0.91	0.95	0.94	0.95
Specificity	0.89	0.36	0.75	0.93	0.84	0.79	0.98	0.86	0.98	0.86
PPV	0.80	0.67	0.73	0.92	0.80	0.82	0.97	0.90	0.97	0.90
NPV	0.75	0.83	0.92	0.62	0.88	0.69	0.94	0.92	0.96	0.92
Precision	0.80	0.67	0.73	0.92	0.80	0.82	0.97	0.90	0.97	0.90
Recall	0.61	0.95	0.91	0.58	0.85	0.74	0.91	0.95	0.94	0.95
F1	0.69	0.78	0.81	0.71	0.82	0.78	0.94	0.92	0.95	0.92

Rad radiomics; DTL deep transfer learning; DLR deep learning radiomics; PPV positive predictive value; NPV negative predictive value.

The calibration curves demonstrate that the Combined model and DLR model achieved the best agreement between predicted probabilities and observed outcomes in both the training cohort (Figure 3C) and test cohort (Figure 3D). These results highlight the superior calibration accuracy of the Combined model and DLR model, reinforcing their reliability for clinical risk prediction.

Through the comparison of multiple models, the DLR signature and the Combined model demonstrated the best overall performance. The DeLong test was used to evaluate the statistical significance of differences between the models. Both the DLR model and the Combined model showed significant improvements over traditional clinical and Rad models in both the training and test cohorts. Additionally, the DLR model exhibited significant improvement over the DTL model in the training cohort; however, while an improvement was observed in the test cohort, it did not reach statistical significance. Notably, the improvement of the Combined model over the DLR model was not statistically significant (Figures 3E,F), which may be due to the limited incremental information gained from incorporating clinical data into the DLR model.

The NRI analysis (Supplementary Figures S3A,B) and IDI analysis (Supplementary Figures S3C,D) demonstrate that the DLR model and the Combined model, which integrate radiomics and deep learning features, consistently outperformed all other models in both the training and test cohorts, highlighting their superior improvement in predictive classification accuracy.

### Clinical use

**Figure 4:**
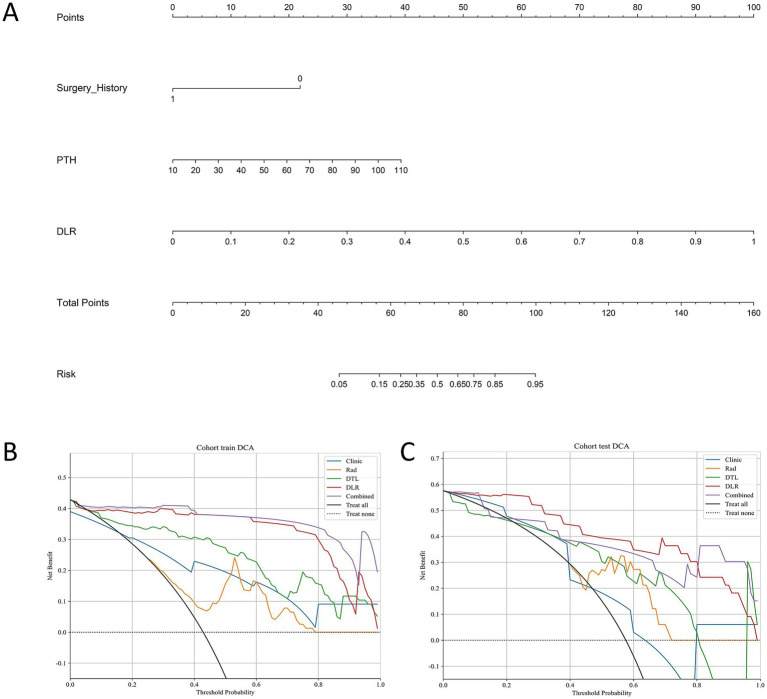
Nomogram and DCA for the combined model. **(A)** Nomogram for the combined model. **(B)** DCA for the training cohort. **(C)** DCA for the testing cohort. DCA decision curve analysis.

As shown in Figure 4, a nomogram was constructed based on Surgery History, PTH, and DLR score to predict patient risk probabilities (Figure 4A). Decision curve analysis (DCA) demonstrated that in both the training cohort (Figure 4B) and the test cohort (Figure 4C), the DLR model and the Combined model consistently provided higher net benefits across the clinically relevant threshold range compared to the clinical model and Radiomics model. These findings indicate that the Combined model and DLR model offer superior clinical utility in MSK stone prediction, providing strong support for clinical decision-making.

## Discussion

The preoperative differentiation of MSK stones from non-MSK multiple kidney stones is essential for optimizing patient management and improving clinical outcomes. MSK is a congenital disease characterized by abnormal development of the renal medullary collecting ducts and papillary ducts, leading to cystic dilations that resemble a sponge-like appearance. First reported by Lenarduzzi in 1939 and later detailed by Cacchi and Ricci (14, 15), MSK is thought to result from mutations or polymorphisms in neurotrophic factor and receptor tyrosine kinase genes during embryonic development (16, 17). The dilated cystic cavities in the collecting ducts lack effective drainage, causing urine retention, secondary infection, and eventual stone formation (4). As stones grow, they detach from the collecting system, often leading to recurrent stone expulsion. Surgical intervention is required for urinary obstruction or recurrent infections, yet anatomical challenges make MSK stone management particularly difficult.

Imaging diagnostics play a critical role in the preoperative evaluation of MSK stones. IVU, traditionally considered the gold standard, reveals characteristic features ranging from a faint blush or linear striations in mild cases to a “bouquet of flowers” appearance in more advanced cases, where cystic dilation of the collecting ducts is evident (15). However, IVU has been largely replaced by CTU due to its high radiation exposure and low sensitivity for detecting small stones. This shift, while improving the overall diagnostic accuracy of kidney stones, has inadvertently led to a decrease in the diagnosis of MSK (15, 18). CTU provides superior diagnostic accuracy for MSK by detecting scattered or clustered hyperdense areas in the renal medulla during the non-contrast phase, clearly distinguishing the corticomedullary region and collecting systems in the arterial phase, and revealing contrast agent accumulation in dilated collecting ducts during the excretory phase (19). However, despite these advantages, its clinical application value in MSK remains limited (20). In patients with early-stage MSK (Forster grade 1 or 2), imaging findings often mimic those of conventional multiple kidney stones, making accurate diagnosis challenging and increasing the likelihood of misdiagnosis. Consequently, the condition is sometimes identified incidentally during endoscopic surgery, which can result in treatment failures or complications.

Recent advances in artificial intelligence, such as deep learning and radiomics, enable the extraction of high-dimensional features from imaging data and offer promising solutions to these challenges (21, 22). For example, Längkvist et al. (23) developed a convolutional neural network-based computer-aided detection system that accurately identifies ureteral stones in thin-slice CT volumes, achieving high sensitivity while minimizing false positives. Similarly, Kim et al. (24) introduced a novel deep learning-based artificial intelligence system for interpreting urolithiasis in CT scans, demonstrating its potential to enhance diagnostic accuracy and efficiency in clinical practice. De Perrot et al. (25) utilized radiomics and machine learning to effectively differentiate kidney stones from phleboliths in unenhanced low-dose CT scans. These studies underscore the transformative potential of AI-driven technologies in improving the detection and management of urinary stones.

In this study, we build upon these advancements by developing a novel DLR signature and Combined model, which integrates radiomics and deep learning features to improve the diagnostic performance of MSK stones. The DLR signature achieved AUCs of 0.964 and 0.962 in the training and test cohorts, respectively, while the Combined model further enhanced these metrics to 0.982 and 0.951. Calibration curves demonstrated satisfying alignment between predicted probabilities and observed outcomes, reinforcing the reliability of the model. NRI and IDI analyses both demonstrated the improved predictive power of the model. Additionally, Grad-CAM visualization provided interpretability, highlighting key imaging regions that influenced model predictions.

Our combined model showed an improvement in AUC compared to the DLR model in the test set, but the difference was not statistically significant. This may be due to the limited variability of the two clinical variables included in the model between the two groups, likely caused by the small sample size. As the sample size increases in future studies, it may be possible to identify more suitable clinical variables to incorporate into the model, thereby enhancing its diagnostic performance.

Despite these promising results, several limitations must be acknowledged. First, the overall sample size was limited, particularly for MSK cases, due to the rarity of the disease. This constraint may affect the statistical power of the analysis and limit the generalizability of the findings. Second, the study was conducted at a single institution, and imaging was performed using a uniform protocol and CT scanner. While this reduces intra-institutional variability, it may limit the applicability of our model across centers with differing imaging protocols and patient demographics. External validation on multi-center datasets with varied acquisition settings is needed for further studies to confirm model robustness. Third, our model relied on manually segmented stone regions and treated each kidney as an independent analytical unit. Although inter-observer segmentation reproducibility was high in a subset of cases (mean Dice coefficient = 0.91), manual delineation remains a potential source of variability. Analyzing each kidney separately—particularly in patients with bilateral stones—may introduce data dependency, potentially inflating model performance. Further studies should consider semi-automated or fully automated segmentation to enhance the reproducibility and reduce labor intensity. Patient-level modeling should also be further investigated.

The proposed models hold significant clinical implications. By seamlessly integrating into clinical workflows, they can enhance the accuracy and efficiency of MSK stone diagnosis, facilitating personalized treatment strategies. Early identification of MSK enables targeted interventions, such as metabolic evaluation and tailored surgical planning, ultimately improving patient outcomes.

## Conclusion

This study presents an innovative approach for diagnosing MSK stones by integrating radiomics and deep learning, achieving high diagnostic accuracy with promising clinical utility. Future research should focus on improving model robustness, expanding dataset diversity, incorporating multi-modal data, and conducting large-sample, multi-center, prospective studies.

## Data Availability

The datasets presented in this article are not readily available because the dataset used in this study is not publicly available and will not be shared due to patient privacy concerns and institutional data protection policies. Further inquiries can be directed to the corresponding author.

## References

[1] Imam TH Patail H Patail H. Medullary sponge kidney: current perspectives. Int J Nephrol Renov Dis. (2019) 12:213–8. doi: 10.2147/IJNRD.S169336, PMID: 31576161 PMC6769051

[2] Carboni I Andreucci E Caruso MR Ciccone R Zuffardi O Genuardi M. Medullary sponge kidney associated with primary distal renal tubular acidosis and mutations of the H+-ATPase genes. Nephrol Dial Transplant. (2009) 24:2734–8. doi: 10.1093/ndt/gfp160, PMID: 19364879

[3] Yagisawa T Kobayashi C Hayashi T Yoshida A Toma H. Contributory metabolic factors in the development of nephrolithiasis in patients with medullary sponge kidney. Am J Kidney Dis. (2001) 37:1140–3. doi: 10.1053/ajkd.2001.24515, PMID: 11382681

[4] McPhail EF Gettman MT Patterson DE Rangel LJ Krambeck AE. Nephrolithiasis in medullary sponge kidney: evaluation of clinical and metabolic features. Urology. (2012) 79:277–81. doi: 10.1016/j.urology.2011.07.1414, PMID: 22014971

[5] Hong Y Xu QQ Huang XB Zhu ZJ Ye HY Zhang FS. Effects of percutaneous nephrolithotomy in the treatment of medullary sponge kidney with calculi. Zhonghua Wai Ke Za Zhi. (2017) 55:742–5. doi: 10.3760/cma.j.issn.0529-5815.2017.10.005, PMID: 29050173

[6] Geavlete P Nita G Alexandrescu E Geavlete B. The impact of modern endourological techniques in the treatment of a century old disease--medullary sponge kidney with associated nephrolithiasis. J Med Life. (2013) 6:482–5. PMID: 24868267 PMC4034299

[7] Forster JA Taylor J Browning AJ Biyani CS. A review of the natural progression of medullary sponge kidney and a novel grading system based on intravenous urography findings. Urol Int. (2007) 78:264–9. doi: 10.1159/000099350, PMID: 17406139

[8] Gillies RJ Kinahan PE Hricak H. Radiomics: images are more than pictures, they are data. Radiology. (2016) 278:563–77. doi: 10.1148/radiol.2015151169, PMID: 26579733 PMC4734157

[9] Wong PK Chan IN Yan HM Gao S Wong CH Yan T. Deep learning based radiomics for gastrointestinal cancer diagnosis and treatment: a minireview. World J Gastroenterol. (2022) 28:6363–79. doi: 10.3748/wjg.v28.i45.6363, PMID: 36533112 PMC9753055

[10] Cui Y Zhang J Li Z Wei K Lei Y Ren J. A CT-based deep learning radiomics nomogram for predicting the response to neoadjuvant chemotherapy in patients with locally advanced gastric cancer: a multicenter cohort study. EClinicalMedicine. (2022) 46:101348. doi: 10.1016/j.eclinm.2022.101348, PMID: 35340629 PMC8943416

[11] Guo L Hao X Chen L Qian Y Wang C Liu X. Early warning of hepatocellular carcinoma in cirrhotic patients by three-phase CT-based deep learning radiomics model: a retrospective, multicentre, cohort study. EClinicalMedicine. (2024) 74:102718. doi: 10.1016/j.eclinm.2024.102718, PMID: 39070173 PMC11279308

[12] Fedorov A Beichel R Kalpathy-Cramer J Finet J Fillion-Robin JC Pujol S. 3D slicer as an image computing platform for the quantitative imaging network. Magn Reson Imaging. (2012) 30:1323–41. doi: 10.1016/j.mri.2012.05.001, PMID: 22770690 PMC3466397

[13] van Griethuysen JJM Fedorov A Parmar C Hosny A Aucoin N Narayan V. Computational Radiomics system to decode the radiographic phenotype. Cancer Res. (2017) 77:e104–7. doi: 10.1158/0008-5472.CAN-17-0339, PMID: 29092951 PMC5672828

[14] Gambaro G Feltrin GP Lupo A Bonfante L D'Angelo A Antonello A. Medullary sponge kidney (Lenarduzzi-Cacchi-Ricci disease): a Padua medical school discovery in the 1930s. Kidney Int. (2006) 69:663–70. doi: 10.1038/sj.ki.5000035, PMID: 16395272

[15] Fabris A Anglani F Lupo A Gambaro G. Medullary sponge kidney: state of the art. Nephrol Dial Transplant. (2013) 28:1111–9. doi: 10.1093/ndt/gfs505, PMID: 23229933

[16] Gambaro G Danza FM Fabris A. Medullary sponge kidney. Curr Opin Nephrol Hypertens. (2013) 22:421–6. doi: 10.1097/MNH.0b013e3283622b86, PMID: 23680648

[17] Ria P Fabris A Dalla Gassa A Zaza G Lupo A Gambaro G. New non-renal congenital disorders associated with medullary sponge kidney (MSK) support the pathogenic role of GDNF and point to the diagnosis of MSK in recurrent stone formers. Urolithiasis. (2017) 45:359–62. doi: 10.1007/s00240-016-0913-6, PMID: 27573101

[18] Xiang H Han J Ridley WE Ridley LJ. Medullary sponge kidney. J Med Imaging Radiat Oncol. (2018) 62:93–4. doi: 10.1111/1754-9485.40_12784, PMID: 30309197

[19] Koraishy FM Ngo TT Israel GM Dahl NK. CT urography for the diagnosis of medullary sponge kidney. Am J Nephrol. (2014) 39:165–70. doi: 10.1159/000358496, PMID: 24531190

[20] Maw AM Megibow AJ Grasso M Goldfarb DS. Diagnosis of medullary sponge kidney by computed tomographic urography. Am J Kidney Dis. (2007) 50:146–50. doi: 10.1053/j.ajkd.2007.03.020, PMID: 17591535

[21] Hosny A Parmar C Quackenbush J Schwartz LH Aerts H. Artificial intelligence in radiology. Nat Rev Cancer. (2018) 18:500–10. doi: 10.1038/s41568-018-0016-5, PMID: 29777175 PMC6268174

[22] Lambin P Rios-Velazquez E Leijenaar R Carvalho S van Stiphout RG Granton P. Radiomics: extracting more information from medical images using advanced feature analysis. Eur J Cancer. (2012) 48:441–6. doi: 10.1016/j.ejca.2011.11.036, PMID: 22257792 PMC4533986

[23] Langkvist M Jendeberg J Thunberg P Loutfi A Liden M. Computer aided detection of ureteral stones in thin slice computed tomography volumes using convolutional neural networks. Comput Biol Med. (2018) 97:153–60. doi: 10.1016/j.compbiomed.2018.04.021, PMID: 29730498

[24] Kim J Kwak CW Uhmn S Lee J Yoo S Cho MC. A novel deep learning-based artificial intelligence system for interpreting urolithiasis in computed tomography. Eur Urol Focus. (2024) 10:3. doi: 10.1016/j.euf.2024.07.003, PMID: 38997836

[25] De Perrot T Hofmeister J Burgermeister S Martin SP Feutry G Klein J. Differentiating kidney stones from phleboliths in unenhanced low-dose computed tomography using radiomics and machine learning. Eur Radiol. (2019) 29:4776–82. doi: 10.1007/s00330-019-6004-7, PMID: 30747299

