# Supplementary material for: Integrated radiomics and deep learning model for identifying medullary sponge kidney stones
Source: Front Med (Lausanne). 2025 Jul 25;12:1623850. doi: 10.3389/fmed.2025.1623850 (PMC12331667; doi:10.3389/fmed.2025.1623850)
Supplement: Supplementary file 1 [file Data_Sheet_1.docx]

**Figure S1. Flowchart of patient selection**

**Figure S2. Feature analysis, model performance, and visualization of radiomics and deep learning features.**(A) Radiomics feature importance.(B) ROC curves for the radiomics model in the training and testing cohort.(C) Deep learning feature importance.(D) ROC curves for the deep learning model in the training and testing cohort. (E) Grad-CAM visualization of deep learning features.

**Figure S3. NRI and IDI analyses for different models in the training and testing cohorts.** (A) NRI in the training cohort. (B) NRI in the test cohort. (C) IDI in the training cohort. (D) IDI in the test cohort. NRI net reclassification improvement, IDI integrated discrimination improvement.
